# Translation, Cross-Cultural Adaptation and Psychometric Validation of the Arabic Version of the Cardiac Rehabilitation Barriers Scale (CRBS-A) with Strategies to Mitigate Barriers

**DOI:** 10.3390/healthcare11081196

**Published:** 2023-04-21

**Authors:** Raghdah Aljehani, Sherry L. Grace, Aseel Aburub, Karam Turk-Adawi, Gabriela Lima de Melo Ghisi

**Affiliations:** 1 Rehabilitation Department, King Abdullah Medical City, Makkah 24246, Saudi Arabia; 2 Faculty of Health, York University, Toronto, ON M3J 1P3, Canada; 3 KITE, Toronto Rehabilitation Institute, University Health Network, Toronto, ON M4G 2V6, Canada; 4 Peter Munk Cardiac Centre, University Health Network, University of Toronto, Toronto, ON M5G 2C4, Canada; 5 Department of Physiotherapy, Applied Science Private University, Amman 11931, Jordan; 6 College of Health Sciences, QU Health, Qatar University, Doha 2713, Qatar

**Keywords:** cardiac rehabilitation, questionnaires and surveys, psychometrics, validity (epidemiology), access to health care, referral

## Abstract

Cardiac rehabilitation (CR) utilization is low, particularly in Arabic-speaking countries. This study aimed to translate and psychometrically validate the CR Barriers Scale in Arabic (CRBS-A), as well as strategies to mitigate them. The CRBS was translated by two bilingual health professionals independently, followed by back-translation. Next, 19 healthcare providers, followed by 19 patients rated the face and content validity (CV) of the pre-final versions, providing input to improve cross-cultural applicability. Then, 207 patients from Saudi Arabia and Jordan completed the CRBS-A, and factor structure, internal consistency, construct, and criterion validity were assessed. Helpfulness of mitigation strategies was also assessed. For experts, item and scale CV indices were 0.8–1.0 and 0.9, respectively. For patients, item clarity and mitigation helpfulness scores were 4.5 ± 0.1 and 4.3 ± 0.1/5, respectively. Minor edits were made. For the test of structural validity, four factors were extracted: time conflicts/lack of perceived need and excuses; preference to self-manage; logistical problems; and health system issues and comorbidities. Total CRBS-A α was 0.90. Construct validity was supported by a trend for an association of total CRBS with financial insecurity regarding healthcare. Total CRBS-A scores were significantly lower in patients who were referred to CR (2.8 ± 0.6) vs. those who were not (3.6 ± 0.8), confirming criterion validity (*p* = 0.04). Mitigation strategies were considered very helpful (mean = 4.2 ± 0.8/5). The CRBS-A is reliable and valid. It can support identification of top barriers to CR participation at multiple levels, and then strategies for mitigating them can be implemented.

## 1. Introduction

Cardiovascular diseases (CVDs) remain a leading contributor to morbidity and mortality worldwide [1]. The burden of CVD is particularly high in countries where Arabic is the official language (i.e., Arabic countries) [2]; globally, this comprises 22 countries. For instance, data from the Eastern Mediterranean (EMR)/the Middle East and North African (MENA) regions—where the majority of countries are comprised of people whose first language is Arabic—show high rates of disability related to CVD, and its incidence is forecasted to grow fastest in these two regions [3,4]. Thus, there is a great need for secondary and tertiary prevention to mitigate the CVD burden in the region.

Cardiac rehabilitation (CR) is such a comprehensive outpatient chronic disease management model with well-established benefits, including reduced hospital admissions and CVD mortality rates [5]. There is evidence demonstrating that CR is effective in Arabic countries as well [6]. Despite these benefits, CR utilization is low around the world [7], including in these regions [8,9].

Barriers are known to exist at the health system, referring provider, program, and patient levels [10]. Grace et al. have developed the Cardiac Rehab Barriers Scale (CRBS) assessing patients’ perceptions of these multi-level barriers to CR enrollment and participation [11,12]. The CRBS has been translated to 17 languages, including 4/5 of the most spoken languages in the world by number of native speakers (Chinese 1.3 billion, Spanish 475 million, English 373 million, and Hindi 344 million) [13]. Arabic is the fourth, with 362 million native speakers around the world [13]. Given the socio-cultural context in Arabic countries, there may be some unique barriers. The scale is key to identifying barriers so they can be mitigated [14]. Indeed, strategies to mitigate top identified barriers could be provided to patients and have recently been expounded.

Accordingly, the aims of this study were to: (a) rigorously translate and cross-culturally adapt the CRBS to Arabic by using the best practices, and then (b) psychometrically validate the translation. This included factor structure, internal reliability, as well as the following forms of validity: face, content, cross-cultural, construct, and criterion. The secondary objectives were to (a) translate and cross-culturally adapt potential strategies to mitigate the barriers, and (b) solicit patient input on their usefulness.

## 2. Materials and Methods

### 2.1. Design

**Figure 1:**
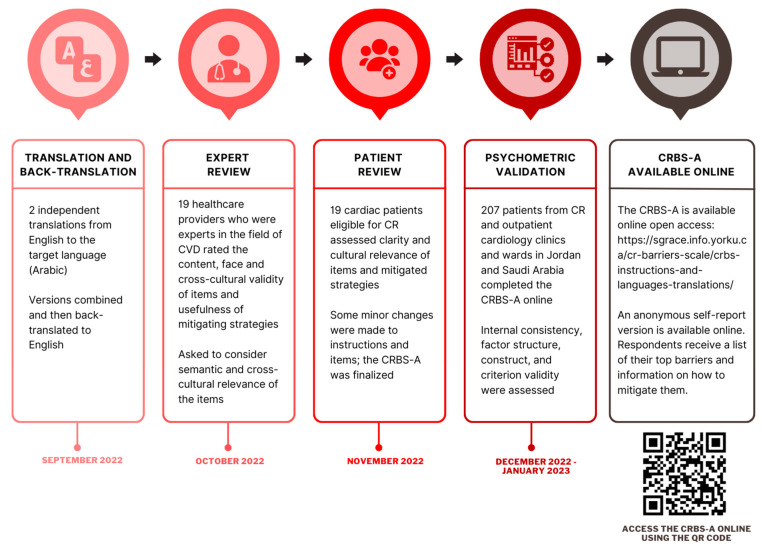
Process of Translating, Adapting and Psychometrically Validating the CRBS-A. Legend: CRBS-A, Cardiac Rehabilitation Barriers Scale-Arabic.

This was a multi-method study comprising (1) translation and cultural adaptation of the CRBS to Arabic using best practices [15], followed by (2) a cross-sectional survey of patients for psychometric validation. Figure 1 displays the multi-step translation, cross-cultural adaptation, and psychometric validation processes (dates provided). The study was approved by the King Abdullah Medical City (22-988; Saudi Arabia) and York University (e2021-013; Canada) research ethics boards.

### 2.2. Materials: Cardiac Rehabilitation Barriers Scale and Barrier Mitigation Strategies

The CRBS is a patient-report survey developed by Grace et al. to assess their perceptions of patient, referring provider, CR program and health system-level barriers to phase II CR enrolment and adherence [11], so reasons for under-use can be understood and addressed [16]. It was developed following an extensive review of the literature, with feedback from cardiologists and CR staff, followed by validation in patient samples; validity and reliability have been established [12]. 

The original English version of the scale is comprised of 21 items, each scored on a 5-point Likert scale (1 = strongly disagree to 5 = strongly agree), as well as an open-ended item for additional barriers. Higher scores indicate greater barriers to CR. The original scale and most translations consist of four subscales: perceived need/healthcare factors; logistical factors; work/time conflicts; and comorbidities/functional status [12]. 

The revised version of the CRBS (i.e., CRBS-R) was used in this study for the first time (https://sgrace.info.yorku.ca/cr-barriers-scale/crbs-instructions-and-languages-translations/, accessed on 8 March 2023), relevant to supervised and unsupervised CR [12]. It was developed following a review of the CRBS literature (45 theses, abstracts, and papers from 25 countries), including psychometric properties, greatest and lowest barriers, as well as additional barriers identified in the studies [12]. Clarifying edits were made to the instructions, and a ‘not applicable’ response option added for each of the items. Item changes primarily involved explication of examples for some of the barriers based on the additional barriers identified in the literature, and re-ordering of items to group related barriers together.

Mitigation responses for each barrier were created by co-author SG, based on a review of quality improvement strategies in the CR field [17] (see Appendix A). Where patients rate a barrier as 4 or 5 out of 5, it is recommended that the mitigation strategy is proffered [12]. These were pilot-tested in English, Simplified Chinese, and Portuguese prior to this study (https://globalcardiacrehab.com/Patients-CRBS, accessed on 8 March 2023). 

### 2.3. Phase 1: Multi-Step Process for Arabic Translation and Cultural Adaptation of CRBS and Associated Barrier Mitigation Strategies

The initial translation of the scale and mitigation strategies from English to the target language (Arabic) was performed by two co-authors (RA and AA) independently; both are fluent in English, and their mother tongue is Arabic. After the two translations had been performed, three co-authors (RA, AA, and KTA) combined and considered the wording. This first version of the instrument was then back-translated into English by co-author KTA, which was reconciled with the merged forward translations by the three Arabic-speaking, bilingual authors, resulting in the second version.

Next, in October 2022, a review committee was formed, comprising a convenience sample of Arabic-speaking healthcare providers who were experts in the field of CVD (including physiotherapists, cardiologists, physiatrists, and family physicians). Contacts from across the EMR were invited to participate via email by the Arabic-speaking co-authors and from the International Council of Cardiovascular Prevention and Rehabilitation community [18]. Using Qualtrics (https://www.qualtrics.com/, accessed on 8 March 2023), they were asked to rate the content validity of items (to enable computation of the content validity index (CVI) for the items (I-CVI) and scale (S-CVI) [19] and usefulness of mitigating strategies. Specifically, for each barrier, respondents were asked ‘yes’ or ‘no’ whether any changes to the item were needed to ensure cross-cultural relevance to Arabic patients, or to improve semantic clarity of the items. If yes, an open-ended description was solicited. They were also asked whether any other barriers should be added. Ratings for mitigation responses ranged from 1 = very unhelpful to 5 = very helpful, with the option to enter open-ended input for each. Edits were made accordingly. 

Next, CR-eligible in or out-patients [20] with a diagnosis of heart disease who were or were not enrolled in CR were purposively-sampled, including to ensure representation of diverse ages, sexes and socioeconomic backgrounds, as well as in clinical presentation. They were asked to review the revised version of the scale and corresponding mitigation strategies in November 2022. Recruitment sources are outlined below. Exclusion criteria were: younger than 18 years old, inability to understand Arabic, and having any significant visual or cognitive condition, or serious mental illness which would preclude their ability to answer the questionnaire. The target sample size was based on the International Society for Pharmacoeconomics and Outcomes Research (ISPOR) guidelines [15], which suggest a minimum of 5–8 respondents.

For this phase, again using Qualtrics, participants were asked to rate clarity of each item from 1 = very unclear to 5 = very clear, or to denote that the item is ‘not applicable’ to them. They were also asked to report any additional barriers. For each corresponding mitigation strategy, participants were asked to rate how useful the response would be if the barrier were applicable to them, on a scale from 1 = not at all useful to 5 = extremely useful. There was also an open-ended option for participants to offer suggestions on how to improve each response. Edits were considered based on responses.

Finally, the back-translated and input-revised version of the Arabic scale was then compared with the original version to consider conceptual discrepancies, with revisions again considered. Overall, this step assessed face, content, and cross-cultural validity of the CRBS-A by multiple stakeholders, and the drafted CRBS-A was ready for psychometric validation.

### 2.4. Phase 2: Cross-Sectional Survey of Patients for Psychometric Validation

#### 2.4.1. Procedure

Participants were recruited in December 2022 and January 2023. Participants gave online consent and completed the survey in Qualtrics. Psychometric properties assessed were factor structure and internal consistency, as well as construct and criterion validity. It was hypothesized that referred patients would have fewer barriers.

#### 2.4.2. Setting and Participants

Inclusion and exclusion criteria for patients are outlined above. Participants recruited for the initial scale review and ultimate psychometric validation were recruited at multiple healthcare centers in Saudi Arabia (where they have CR programs) and in Jordan (where there is no CR, although patients have access to physical therapy), given CR is not pervasive in Arabic-speaking countries [8]. Centers in Saudi Arabia were King Abdullah Medical City (Makkah), Al Noor Hospital (Makkah), and Prince Sultan Cardiac Centre (Riyadh). Centers in Jordan were the King Abdullah University Hospital (Irbid) and Albasheer Hospital (Amman). The patient inclusion and exclusion criteria from step 1 applied. For the psychometric validation phase, to ensure adequate sample size for the factor analysis, 10 patients were sought per item [21], or in this case, 210 participants.

#### 2.4.3. Measures

Sociodemographic characteristics (e.g., age, sex, socioeconomic status, social support) were self-reported using investigator-generated items with forced-choice response options. To assess criterion validity, if they were referred to CR (yes/no) was collected via self-report as well.

Upon completing the survey, corresponding mitigating strategies were provided to patients for any barrier scored ≥ 4/5. After reviewing them, participants were asked how helpful these were on a Likert-type scale ranging from 1 = very unhelpful to 5 = very helpful.

### 2.5. Data Analysis

The Statistical Package for Social Sciences v.28 (SPSS Inc., Chicago, IL, USA) was used for all data analysis. Descriptives of responses from the expert and patient review were examined by the co-authors, with edits made to items as applicable as outlined above. 

For the patient validation survey, first, a descriptive analysis of participant characteristics was performed. Next, exploratory factor analysis (EFA) was undertaken. Factor extraction was conducted using the principal component method, with varimax rotation. The number of factors extracted was determined by considering those with eigenvalues ≥ 1.0, percentage of variance accounted for, and examination of the Scree plot. Item factor loadings ≥ 0.3 were considered in finalizing the items for each factor and interpreting them.

A descriptive examination was performed of each CRBS item. Where participants completed more than 80% of the items, a mean total CRBS score was computed. Subscale scores were also computed based on the results of the factor analysis. 

Next, internal consistency was determined by calculating Cronbach’s *alpha* values of the scale and subscales. *Alpha* ≥ 0.70 was considered acceptable, reflecting the correlation of the items among themselves and with the total score [21]. The scale’s Cronbach’s alpha reliability coefficient for internal consistency if the individual item is removed from the scale was also checked.

The CRBS was not normally distributed. Therefore, to assess construct validity, Spearman’s correlation, Wilcoxon tests, and Kruskal–Wallis tests were applied to explore associations between sociodemographic characteristics of study participants and their CRBS scores, given associations between CR use and social determinants of health [22]. Finally, to consider criterion validity, differences in total CRBS scores by CR referral were tested with Wilcoxon tests. Note that given there was no available CR in Jordan and the number of tests which can inflate error, construct validity assessments and of the association between the CRBS items and CR enrolment were undertaken on an exploratory basis only. The level of significance for all tests was set at 0.05. 

## 3. Results

The translation and cultural adaptation process as well as psychometric validation proceeded as planned. This is outlined below. 

### 3.1. Translation and Cultural Adaptation

Following translations and harmonization of the CRBS to Arabic, the 19 expert health professionals deemed all 21 items in the original CRBS version applicable to the Arabic context. For items 1 and 3, two respondents suggested changes; for item 2, three suggested changes, and for items 6, 7, and 20, one respondent each suggested change. Minor changes to the instructions and in items 1, 3, 5, and 6 were made based on these comments from experts (Appendix B). The I-CVIs ranged from 0.8 to 1.0, and the S-CVI was 0.9, which establishes that the Arabic version of CRBS has acceptable content validity.

Helpfulness ratings of barrier mitigation responses ranged from 4.1 to 4.4/5 (mean 4.3 ± 0.1). Given this and in reviewing the open-ended input, only minor edits were made to mitigation strategy responses. 

For the patients’ input, 19 reviewed the CRBS-A items and responses. Clarity of items ranged from 4.3 to 4.7/5 (mean 4.5 ± 0.1). The number of respondents selecting “not applicable” for each item ranged from 1 to 3 (i.e., maximum 16% of respondents). In addition, considering open-ended input, no further changes were made to the CRBS-A at this stage. The final CRBS-A is shown in Appendix B.

Patient helpfulness of response ratings ranged from 4.3 to 4.7/5 (mean 4.5 ± 0.1). Given the input (which included comments such as “very clear”), no edits were made to mitigation strategy responses (Appendix A).

### 3.2. Psychometric Validation

The sample was comprised of 207 participants, of which 49 (23.7%) participated in CR. Most patients were male, retired, with a mean age of 58, and with 13 years of formal education (Table 1).

The structure of the scale was first assessed using EFA. The Kaiser–Meyer–Olkin value was 0.875, and Bartlett’s test was significant (chi-square 2270.417; *p* < 0.001), which indicated data suitability for factor analysis. Four components with eigenvalues ≥ 1.0 were obtained. These factors, considered together, accounted for 62.7% of the total variance. Table 2 displays the factor structure of the CRBS-A, including item loadings.

The first factor had 8 items and reflects time conflicts and a lack of perceived need/excuses. The second factor (5 items) reflects preference to self-manage. The third factor (3 items) reflects logistical barriers. The fourth factor (5 items) reflects health system issues and comorbidities. The first three factors of the Arabic version of the CRBS showed good internal consistency (Cronbach′s *alpha* ≥ 0.7; Table 2). However, the alpha for factor 4 fell short of the 0.7 threshold. Overall, however, as the α for the total CRBS was 0.90, the internal consistency of the scale is supported.

As shown in Table 1, with regard to construct validity, there was only a trend for an association between total barriers and greater worry related to healthcare costs. With regard to criterion validity, significant associations were observed between total scores and being referred to CR.

Finally, top barriers for those that enrolled in CR were “distance”, “difficulties in accessing sessions that require attendance in person”, and “it took too long to get referred and into the program”. Top barriers for those that did not were “I did not know about cardiac rehab”, “my doctor did not feel it was necessary”, and “difficulties in accessing sessions that require attendance in person”. Exploratory analyses were performed to examine CRBS-A item, subscale, overall scores by CR enrolment. Respondents who enrolled in CR had significantly lower scores on 4/21 items (cost, not knowing about CR, doctor not feeling it was necessary, many people with heart problems do not go and they are fine) and higher on 6/21 items (already exercising at home, severe weather, travel, time constraints, work responsibilities, too long to get referred) than those who did not. While there was no difference in total scores by enrolment status, factor 4 subscale scores (i.e., health system issues, comorbidities) were significantly greater in those who did not enroll (*p* = 0.04), with a trend for factor 3 (logistical barriers; *p* = 0.08).

### 3.3. Main Barriers and Usefulness of Strategies to Mitigate These Barriers

CRBS item scores ranged from 2.2 to 4.1/5 (Table 3). The top CR barriers were “I did not know about cardiac rehab”, “my doctor did not feel it was necessary”, “distance”, and “difficulties in accessing sessions that require attendance in person”. Items with the highest number of participants indicating that the barrier was not applicable were: “other health problems prevent me from going” (16% of respondents), “work responsibilities” (15%), and “I prefer to take care of my health alone, not in a group” (14%). Other barriers cited by participants included no knowledge of what CR is and not having CR centers close to home, which are already included items (items 5 and 1, respectively).

Overall, the mean usefulness of the information provided to mitigate the barriers was rated 4.2 ± 0.8/5; 85% of respondents found the information to be useful or very useful.

## 4. Discussion

Following best practices, this study has rigorously translated, cross-culturally adapted, and psychometrically validated the Arabic version of the CRBS scale. Through this process, all 21 items of the scale were retained, with revisions made to 4 items to improve clarity based on expert and patient reviews. Face, content, and cross-cultural validity were supported. Subsequent exploratory factor analysis revealed four factors: time conflicts, lack of perceived need and excuses; preference to self-manage; logistical problems; as well as health system issues and comorbidities. Even in the EMR context of low CR availability, criterion validity was confirmed by the significantly lower mean CRBS scores in patients who were referred to CR than those who were not. Overall, these results confirm the validity and reliability of the CRBS-A in EMR settings with and without accessible CR. This study has also established for the first time mitigation strategies for each barrier (Appendix A), which patients rated as highly useful. 

It is important to compare and contrast this CRBS translation with others. In fact, this is the 18th translation of the CRBS; among all versions, there are seven that also comprise four factors. However, the items that loaded on each factor were somewhat different, potentially due to the fact that the translation is based on the CRBS-R. The internal consistency of the Arabic version was 0.90, which is one of the highest when compared to all other CRBS translations, again potentially due to the fact that it is based on the CRBS-R. The reliability of some subscales in seven of the 18 CRBS translations was below 0.70 [21], similar to factor 4 in this Arabic translation; this is likely due to the fact that some barriers are quite unique. The reader is referred to the review of all CRBS translations for more information [12]. 

Mean scores were comparable to other samples which consist of many non-enrollees [12], as well as with other samples from the EMR [9,23,24]. Indeed, the CRBS has now been administered in four EMR countries (i.e., Qatar [9], Iran [23,24], Saudi Arabia, Jordan), establishing that it is valid in the socio-religious context as well. The CRBS has been administered in other countries with very low CR availability [25] such as Brazil [26,27,28,29,30,31], Colombia [32,33,34], Iran [23,24], Czechia [35], Indonesia [36], China [37,38,39,40], Malaysia [41,42], and South Korea [43,44], again supporting its applicability globally. This may have been the first study however where the CRBS has been administered in a context without available CR, which did render it infeasible to fully assess construct and criterion validity.

### 4.1. Implications

The CRBS-A is available in anonymous patient-report format online to use open access, with ethics approval: https://globalcardiacrehab.com/Patients-CRBS, accessed on 8 March 2023. Besides the Arabic version, three other languages are also available: English, Portuguese, and Simplified Chinese. After completing the survey, respondents are presented with a list of their top barriers along with the validated and useful suggestions on how to mitigate them, with links to resources in their own language. Respondents can save or print their top barriers or discuss them with healthcare providers. Future research is needed to test if mitigation strategies have an impact on CR utilization using a randomized, controlled, and prospective study design.

As evidenced by the top barriers, structural changes are needed to augment CR utilization; mitigating many of the barriers is beyond the control of patients, such that it is incumbent upon the professional community to mitigate them. Chiefly again, there is a dire need for more CR programs [25], and reimbursement of CR care [45] so that patients can actually be informed about and referred to available programs. For example, satellite CR programs are being initiated in Qatar [46]. Acute cardiac care physicians would then need to be educated about the benefits of CR and the importance of referral [47,48], with the setup of automatic inpatient referral [49,50] so patients do not perceive that their providers consider CR unnecessary [51]. Finally, the 3rd and 4th top barriers were distance and relatedly challenged to in-person sessions, which can be mitigated by hybrid session delivery within CR programs [52]. 

### 4.2. Limitations

While experts from across the EMR gave input, data were only collected from patients in two countries; thus, results may not be generalizable to all Arab-speaking settings. Second, there were multiple comparisons, which can lead to inflated error; the item-level CRBS analysis for criterion validity was solely exploratory and as such, caution is warranted in interpreting the results regarding association to CR enrolment. Third, physical activity history was not assessed using a validated scale. Fourth, causal conclusions cannot be drawn. Finally, future research is recommended to again assess these psychometric properties, and also test others such as test–retest reliability. 

## 5. Conclusions

The CRBS is the only available tool designed to identify cardiac patients’ multi-level barriers to CR enrolment and participation. The Arabic version of this instrument—CRBS-A—was developed and validated in this study. While more research is needed, overall results confirmed good psychometric properties, which supports its administration. Moreover, for the first time, this study has proffered strategies for mitigating them, which providers and patients alike established as helpful. In conjunction with needed health system changes, it is hoped that this freely-available online resource will support mitigation of CR barriers at the patient-level, so ultimately more patients in Arab-speaking countries and beyond reap the life-saving benefits of CR.

## Figures and Tables

**Table 1:** Characteristics of validation study participants, and by CRBS-A score (N = 207).

Sociodemographic Characteristics	n (%)/ Mean ± SD	Median CRBS-A Score (Interquartile Range)	*p* ^a^
Age	58.2 ± 13.9	-	0.62
Less than 65 years old	123 (59.4)	2.8 (2.3–3.5)	
65 years old or older	66 (31.9)	2.9 (2.6–3.3)	
Sex			0.27
Male	128 (61.8)	2.9 (2.5–3.4)	
Female	75 (36.2)	2.8 (2.4–3.3)	
Other/prefer not to answer	2 (1.0)		
Work status			0.33
Retired	93 (44.9)	3.0 (2.6–3.4)	
Full or part-time	59 (28.5)	2.7 (2.4–3.5)	
Unemployed	27 (13.0)	2.9 (2.3–3.5)	
Disability/sick leave/modified duties	17 (8.2)	2.7 (2.0–3.1)	
Looking to get a paid job	8 (3.9)	2.7 (2.6–3.3)	
Social support (/5) ^b^	3.3 ± 1.1	-	0.26
Definitely	32 (15.5)	3.1 (2.6–3.5)	
Most of the time	46 (22.2)	3.0 (2.5–3.5)	
Sometimes	78 (37.7)	2.8 (2.4–3.3)	
Rarely	34 (16.4)	2.9 (2.6–3.3)	
Never	13 (6.3)	2.5 (2.1–3.0)	
Years of formal education	13.2 ± 6.2	-	0.66
Less than 8 years	18 (8.7)	2.8 (2.4–3.6)	
8 or more years	152 (73.4)	2.7 (2.4–3.3)	
Worrying about having enough money to pay for health care			0.20
I often worry	65 (31.4)	2.8 (2.5–3.3)	
I worry sometimes	105 (50.7)	3.0 (2.6–3.5)	
I never worry	35 (16.9)	2.7 (2.3–2.9)	
Physical activity prior to heart problem			0.58
Yes	40 (19.3)	2.7 (2.4–3.6)	
No	159 (76.8)	2.9 (2.5–3.4)	
Referral to CR			0.04
Yes	43 (20.8)	2.6 (2.3–3.3)	
No/I do not know	158 (76.3)	3.1 (2.5–3.4)	

CR: cardiac rehabilitation; CBBS-A: Cardiac Rehab Barriers Scale Arabic; SD: standard deviation. a. Spearman’s correlation, Wilcoxon or Kruskal–Wallis test, as appropriate. b. responses were also scored on a Likert scale from 1 “never” to 5 “definitely” so a mean score could be computed.—not applicable.

**Table 2:** Exploratory factor analysis of the CRBS-A and reliability of factors, N = 207.

Item	Factor 1: Time Conflicts/Lack of Perceived Need/Excuses	Factor 2: Prefer to Self-Manage	Factor 3: Logistical Barriers	Factor 4: Health System Issues/Comorbidities
11. …of time constraints (e.g., too busy, inconvenient class time)	0.799			
7. …I already exercise at home, or in my community	0.768			
12. …of work responsibilities	0.741			
10. …travel (e.g., holidays, business, cottage)	0.688			
6. …I do not need cardiac rehab (e.g., feel well, heart problem treated, not serious)	0.676			
8. …severe weather	0.674			
9. …I find exercise tiring or painful	0.480			
4. … family responsibilities (e.g., caregiving)	0.434			
18. … I can manage my heart problem on my own		0.840		
17. … many people with heart problems do not go, and they are fine		0.824		
19. … I think I was referred, but the rehab program did not contact me		0.786		
21. …I prefer to take care of my health alone, not in a group		0.604		
15. …I am too old		0.572		
3. … difficulties in accessing sessions that require attendance in person (for example, lack of car or suitable transportation)			0.818	
2. … costs (e.g., program participation costs, transportation and parking costs, qualification requirements such as shoes, exercise, equipment/educational materials, equipment costs)			0.725	
1. … distance (e.g., there is not a program in the same area, too far for travel)			0.661	
5. …I did not know about cardiac rehab (e.g., doctor did not tell me about it)				−0.711
16. …my doctor did not feel it was necessary				−0.597
14. …other health problems prevent me from going.				0.581
20. …it took too long to get referred and into the program				0.576
13. …I do not have the energy				0.413
Variance explained	23.3%	17.8%	12.7%	8.9%
Eigenvalues	7.50	2.41	2.03	1.25
Reliability	0.88	0.84	0.78	0.50

**Table 3:** CRBS-A mean item, factor, and total scores.

CRBS-A Item	Total (N = 207)	N/A (%)
1. …distance (e.g., there is not a program in the same area, too far for travel)	3.4 ± 1.5	21 (10.1)
2. …costs (e.g., program participation costs, transportation and parking costs, qualification requirements such as shoes, exercise, equipment/educational materials, equipment costs)	3.2 ± 1.5	19 (9.2)
3. …difficulties in accessing sessions that require attendance in person (for example, lack of car or suitable transportation)	3.4 ± 1.5	20 (9.7)
4. …family responsibilities (e.g., caregiving)	3.1 ± 1.5	20 (9.7)
5. …I did not know about cardiac rehab (e.g., doctor did not tell me about it)	4.1 ± 1.3	7 (3.4)
6. …I do not need cardiac rehab (e.g., feel well, heart problem treated, not serious)	2.6 ± 1.3	14 (6.8)
7. …I already exercise at home, or in my community	2.6 ± 1.4	17 (8.2)
8. …severe weather	2.8 ± 1.4	16 (7.7)
9. …I find exercise tiring or painful	2.9 ± 1.4	13 (6.3)
10. …travel (e.g., holidays, business, cottage)	2.3 ± 1.5	17 (8.2)
11. …of time constraints (e.g., too busy, inconvenient class time)	2.8 ± 1.4	20 (9.7)
12. …of work responsibilities	2.5 ± 1.6	31 (15.0)
13. …I do not have the energy	2.9 ± 1.4	15 (7.2)
14. …other health problems prevent me from going.	2.2 ± 1.4	33 (15.9)
15. …I am too old	2.7 ± 1.5	21 (10.1)
16. …my doctor did not feel it was necessary	3.6 ± 1.5	10 (4.8)
17. …many people with heart problems do not go, and they are fine	2.6 ± 1.4	17 (8.2)
18. …I can manage my heart problem on my own	2.8 ± 1.4	11 (5.3)
19. …I think I was referred, but the rehab program did not contact me	2.5 ± 1.4	17 (8.2)
20. …it took too long to get referred and into the program	2.5 ± 1.6	28 (13.5)
21. …I prefer to take care of my health alone, not in a group	2.8 ± 1.4	28 (13.5)
Factor 1: time conflicts/lack of perceived need/excuses	2.7 ± 1.0	-
Factor 2: prefer to self-manage	2.7 ± 1.0	-
Factor 3: logistical barriers	3.3 ± 1.3	-
Factor 4: health system issues/comorbidities	3.1 ± 0.8	-
Total CRBS-A	2.9 ± 0.8	-

N/A, not applicable; CRBS-A, CR Barriers Scale-Arabic, CR, cardiac rehabilitation. Note: CRBS-A scores range from 1–5, with higher scores indicating greater barriers. Mean ± standard deviation shown.

## Data Availability

Data available on request due to ethical restrictions.

## Appendix A. Mitigation Responses for Each Barrier

How to Overcome Your Cardiac Rehab Barriers

Here are your top cardiac rehabilitation barriers and ideas on how to overcome each:

1. Distance

Check with your cardiac rehab program to ask if there is a closer program to your home or work. If there is not, you can also ask about home-based cardiac rehab options. It may be possible for you to get your cardiac rehab through phone or video calls with your cardiac rehab team, after going for a few sessions to become oriented. If you do not have a contact at the cardiac rehab program, ask your primary care provider to find out if there is a closer program for you to attend. If there is no program close by or the program cannot support you to do your rehab from home, we suggest you: (1) have an appointment with a physiotherapist if possible to develop an exercise plan, and then you could follow along here from home https://www.bhf.org.uk/informationsupport/support/cardiac-rehabilitation-at-home/cardiac-rehabilitation-exercise-videos, accessed on 8 March 2023, (2) work with your primary care provider to make sure all of your heart risk factors are controlled (i.e., cholesterol, blood pressure, tobacco cessation supports, healthy diet, psychosocial management), and (3) complete the comprehensive heart patient education program available free online here: https://www.healtheuniversity.ca/en/cardiaccollege/Pages/default.aspx, accessed on 8 March 2023. There is a 12-week challenge there as well you may want to try (see Thrive on right).

2. Cost

Some cardiac rehab programs cost money to attend, and others do not. Ask your cardiac rehab team about the cost to attend the program. If there is no free option available and you have financial pressures, the cardiac rehab team may be able to reduce the cost. You could also ask your primary care provider about any available programs with no cost. Alternatively if you truly cannot afford cardiac rehab and there are no low-cost options available, we suggest you: (1) have an appointment with a physiotherapist if possible to develop an exercise plan, and then you could follow along here from home https://www.bhf.org.uk/informationsupport/support/cardiac-rehabilitation-at-home/cardiac-rehabilitation-exercise-videos, accessed on 8 March 2023, (2) work with your primary care provider to make sure all of your heart risk factors are controlled (i.e., cholesterol, blood pressure, tobacco cessation supports, healthy diet, psychosocial management), and (3) complete the comprehensive heart patient education program available free online here: https://www.healtheuniversity.ca/en/cardiaccollege/Pages/default.aspx, accessed on 8 March 2023. There is a 12-week challenge there as well you may want to try (see Thrive on right).

If your concerns are related to costs for transportation, again, ask your cardiac rehab team if there are lower cost parking passes available, public transit, and/or support to cover transportation costs.

3. Transportation Problems

Check Google Maps for different ways to get to your cardiac rehab program (e.g., car, public transit). Ask the cardiac rehab team if there are supports available or if you can arrange to carpool. Ask if an extended family member could get you to some sessions.

If there is no support available to help you get to sessions, you can also ask about home-based cardiac rehab options. It may be possible for you to get your cardiac rehab through phone or video calls with your cardiac rehab team, after going for a few sessions to become oriented. If this is an option, inquire about the cost.

If you do not have a contact at the cardiac rehab program, ask your primary care provider to find out for you if there is a closer program for you to attend.

If there is no support for you to get to cardiac rehab and no home-based option, we suggest you: (1) have an appointment with a physiotherapist if possible to develop an exercise plan, and then you could follow along here from home https://www.bhf.org.uk/informationsupport/support/cardiac-rehabilitation-at-home/cardiac-rehabilitation-exercise-videos, accessed on 8 March 2023, (2) work with your primary care provider to make sure all of your heart risk factors are controlled (i.e., cholesterol, blood pressure, tobacco cessation supports, healthy diet, psychosocial management), and (3) complete the comprehensive heart patient education program available free online here: https://www.healtheuniversity.ca/en/cardiaccollege/Pages/default.aspx, accessed on 8 March 2023. There is a 12-week challenge there as well you may want to try (see Thrive on right).

4. Family Responsibilities

We know family comes first and you have obligations to help your loved ones, but remember if you want to continue your family responsibilities for as long as possible, it is important for you to put your heart health first right now. If you make time for cardiac rehabilitation, you will be less likely to need to go to the hospital again, and you will also become stronger and more able to do things needed to help your family. People who go to cardiac rehab live longer. If you discuss this with your family, I am sure they will agree you need to find ways to make it to your cardiac rehab sessions.

Here, are some ideas:

- Ask extended family members to take over some of your family responsibilities for a short period.
- Some cardiac rehab programs welcome family members into the program. If your family member is an adult that can do some basic walking, they may be able to join in and reap the benefits as well. Ask your program.
- See if you can hire someone (e.g., teen neighbor, personal support worker, cleaning help) to take over some of your family responsibilities in the short term.
- Check with your primary care provider for available community services for caregiving in the home/respite.
- Ask your cardiac rehab program about home-based options. It may be possible for you to get your cardiac rehab through phone or video calls with your cardiac rehab team, after going for a few sessions to become oriented.

5. Not knowing about cardiac rehabilitation

It is unfortunate you were not encouraged to attend cardiac rehab by your healthcare providers. They are often very busy, so they may have overlooked it. We are so glad that you have learned about cardiac rehab and are taking this self-assessment.

You can find out more about what cardiac rehab offers here: https://www.youtube.com/watch?v=bjiOaz-0H2E, accessed on 8 March 2023. Participating in cardiac rehab results in many benefits, such as better quality of life, fewer repeat visits to the hospital and need for heart procedures; furthermore, patients who go to cardiac rehab live longer.

Please ask your primary care provider about getting a referral, and/or your cardiac specialist. Then, the program will contact you about getting started and answer any questions you might have. You could share this barrier self-assessment with them! If you are still having trouble getting a referral, you can contact us at the Gmail address below.

6. I do not need cardiac rehab

You may feel ok and “cured” from bypass surgery or getting a stent. It is important for you to know that the blood vessels throughout your body are still diseased. Patients who have heart disease are at higher risk of having another heart problem than people without heart disease, but you can lower your risk through participating in cardiac rehab. It results in many benefits, such as better quality of life, less repeat visits to the hospital and need for heart procedures. Moreover, patients who go to cardiac rehab live longer.

Additionally, know that your cardiac rehab program will be tailored to you. The rehab staff will focus on your goals and create a plan just for you to get back to your best health. Cardiac rehab is a very comprehensive program that considers not only medical risk factors such as cholesterol and blood pressure, but also supports you to live a heart-healthy lifestyle (e.g., exercise, diet, tobacco cessation), provides education, and support for returning to your life roles and for optimizing your psychosocial well-being.

Cardiac rehab is recommended for all heart patients in medical guidelines because of all these benefits. If you have not been referred, ask your primary care provider at your next opportunity.

7. Already exercising at home/community

That is excellent that you are active. That is a big part of reducing your risk of having further heart problems. However, cardiac rehab is not just about exercise, so you need to go to reap all the benefits. The cardiac rehab staff will certainly want to hear about your successes in incorporating exercise into your life and encourage you to continue that. They will also check your cholesterol and blood pressure control, provide any needed information to support you in eating a heart-healthy diet, assess your psychosocial well-being and help you where needed, and assist you in returning to all your life roles after your heart event.

Given how comprehensive cardiac rehab is, participation results in many benefits, such as better quality of life (you can get back to doing all the things you love and feel great while doing it), less repeat visits to the hospital and need for heart procedures, and patients who go to cardiac rehab live longer. If you have not yet been referred, ask your primary care provider at your next opportunity.

8. Severe weather

We definitely want you to arrive at cardiac rehab safely. If road conditions are poor due to weather, you may want to call your cardiac rehab program contact to ask about doing your session remotely (e.g., phone or videocall). Work with your cardiac rehab program to identify a place to get your exercise inside away from the elements.

9. Exercise is tiring and painful

Many patients find exercise tiring at the beginning, and may have memories of being tired from exercise in the past. However, at cardiac rehab, the team will develop an exercise program just for you, so you will not be as tired. If you do find yourself too tired, let the staff know, and they will work with you to optimize your exercise routine. Research shows that you will actually have more energy after exercising. Most of our cardiac rehab patients tell us they cannot believe how much better they feel once they get going with their exercise program.

Other patients find exercise painful due to musculoskeletal issues or other health conditions such as arthritis. Your cardiac rehab team will talk to you about non-weight bearing exercise options (e.g., recumbent bikes, pool) and how to relieve pain. They will develop an exercise plan specific to you that takes into consideration any joint or other pain you may have.

10. Travel

No one can know when heart disease will strike! After your heart event, we know you want to get back to your life activities. Remember however that cardiac rehab is a standard of care for heart patients just like a stent and heart medications, because of all the research showing its’ positive impact. Participating in cardiac rehab results in many benefits, such as better quality of life, less repeat visits to the hospital and need for heart procedures, and patients who go to cardiac rehab live longer.

So, to be able to travel for many years to come, it is important to actively participate in your cardiac rehab program now. It may be possible to get started with your program in person, and then move to more remote delivery, where your cardiac rehab team supports you through your program via phone or videocalls for example. Ask at your program what could be done so you can complete your full program.

If you absolutely cannot get to sessions, you could do follow this online cardiac rehab exercise routine https://www.bhf.org.uk/informationsupport/support/cardiac-rehabilitation-at-home/cardiac-rehabilitation-exercise-videos, accessed on 8 March 2023, and complete the comprehensive heart patient education program available free online here: https://www.healtheuniversity.ca/en/cardiaccollege/Pages/default.aspx, accessed on 8 March 2023. Make sure you work with your doctor to ensure all your heart risk factors are controlled through medication.

11. Time constraints

We know how busy modern life is. It is very important however you make time for cardiac rehab, as you will end up having more energy to do the things you need to do, and you will live longer.

Here are some suggestions to consider:

- Talk to your cardiac rehab team about session time availability to find something that works for you.
- Some programs have home-based supports, so you exercise when you can at home or in your community but have regular contacts with the cardiac rehab staff to address all your heart risk factors and educate you about your heart health via phone or videocall at your convenience.
- Talk to your cardiac rehab program contact about what parts of the program are most important for you, and how you can fit it in.
- Most programs offer stress management, which includes a time management component. This can help you get a better hold of you
- Remember you do not need to do all your exercise at once; you can do 10 min here and there each day.
- See if family members or others can take some of your load while you are doing your cardiac rehab program. Your loved ones want to see you well and have you around for many more years to come.

12. Work responsibilities

Many heart patients do not have sick benefits or the option of modified work or work hours through their recovery. It would be worthwhile to talk to your cardiac rehab program contact to determine if there is session availability before or after your work hours.

If there is not, you can also ask about home-based cardiac rehab options. It may be possible for you to get your cardiac rehab through phone or video calls with your cardiac rehab team, after going for a few sessions to get oriented.

If the program cannot offer rehab in a way that works with your work schedule, we suggest you: (1) follow this heart exercise program online https://www.bhf.org.uk/informationsupport/support/cardiac-rehabilitation-at-home/cardiac-rehabilitation-exercise-videos, accessed on 8 March 2023, (2) work with your primary care provider to make sure all of your heart risk factors are controlled (i.e., cholesterol, blood pressure, tobacco cessation supports, healthy diet, psychosocial management), and (3) complete the comprehensive heart patient education program available free online here: https://www.healtheuniversity.ca/en/cardiaccollege/Pages/default.aspx, accessed on 8 March 2023. There is a 12-week challenge there as well you may want to try (see Thrive on right).

13. Not having energy

Many heart patients do not feel well, and they are also often down due to trying to cope with their heart disease. You are not alone. Your cardiac rehab team will understand this and support you.

Given the benefits of participation, and that cardiac rehab programs are individually tailored to meet your needs, we recommend you try it out and start. Be open with your cardiac rehab program contact about your lack of energy and how it could be a barrier to you fully engaging in the program. You can work together to focus on only your key goals and start slow. Make a plan to re-visit how you are feeling after a couple of weeks, to see if you can continue on or something needs to change.

Participating in cardiac rehab will result in you having more energy. They can also assess your psychosocial well-being and help you if there are non-heart-related reasons for your low energy.

14. Other health problems

Almost every cardiac rehab participant has a health problem other than heart disease. Cardiac rehab staff are ready to support you to improve your heart risk factor control, while taking into consideration your overall health status. Here are some examples:

- For patients with diabetes, the rehab team will teach you about eating timing in relation to exercise, help you to assess your blood sugar, and teach you about caring for your feet.
- For patients with arthritis, the rehab team can suggest non-weight-bearing exercise and work with you to achieve pain control.
- For patients with osteoporosis, your rehab team will assess you for your risk of a fall, and put extra safety measures in place where needed.

Remember all these other health conditions are also ameliorated with exercise, and following a healthy diet helps too. So these other health problems are actually extra reasons why you should participate in cardiac rehab.

15. Too old

There is no upper age limit to cardiac rehab participation. In fact, there is a lot of research showing how beneficial cardiac rehab is in older people. If you need support with transportation, or have sensory limitations, talk to the program staff regarding what accommodations can be made to enable you to fully participate.

16. Doctor did not feel it was necessary

It is unfortunate you perceived one of your healthcare providers was not supportive of your participation in cardiac rehab. This is likely an error, because based on a lot of excellent research showing its benefits, doctors are directed to refer all their heart patients to cardiac rehab. Perhaps your doctor was not very familiar with what cardiac rehab entails. It is a comprehensive program to provide you education regarding your heart and how to manage your condition (including the importance of the pills your doctor prescribes), supports you in making heart-healthy behavior changes, as well as in returning to your life roles.

You can get referred to CR by any physician. So you could ask your cardiologist if you have one or primary care physician to refer you. Some programs accept referrals from patients or other providers. It is so important to your health, so be sure to get referred soon.

17. I do not think it will help me

Actually, very rigorous research shows that participating in cardiac rehab results in many benefits, such as better quality of life (and you can feel stronger and able to get back to the activities you want to engage in), less repeat visits to the hospital and need for heart procedures, and patients who go to cardiac rehab live longer. For this reason, it is recommended that all heart patients go to cardiac rehab. If you participate in cardiac rehab, you will likely live many more years than the patient that does not go.

18. Manage own heart problem

We are glad that you take an active stance towards managing your health. Indeed, in cardiac rehab we encourage that. We want to help alongside you.

Cardiac rehab is very comprehensive. Most heart patients are not familiar with how their heart and circulation works, what their pills do, why they need to control their blood pressure and cholesterol, etc. In cardiac rehab we also support patients to optimize their psychosocial well-being and return to life roles. So while you can likely manage some of these elements alone, there are usually some areas where our knowledgeable staff can really help patients.

While you can get quite a bit of information about managing your heart health online, you will not get information specific to your health situation or have someone to answer your specific questions.

You might consider getting started in a program to see what it is all about. You can tell the cardiac rehab staff that you would prefer to be a bit independent in your recovery, and they will certainly accommodate and support that.

If you are a bit of an introvert like me, I can see you might not want to make the effort to go in and start a program. However, research shows that participating in cardiac rehab results in many benefits, such as better quality of life, less repeat visits to the hospital and need for heart procedures, and patients who go to cardiac rehab live longer than those who do not.

19. I am not interested or motivated

You may have gotten a stent or other procedure for your heart, and while these are lifesaving, unfortunately they are not cures. Heart disease is a chronic condition, and you are at higher risk of having another heart event compared to people without heart disease.

Participating in cardiac rehab has many benefits, such as better quality of life, less visits to the hospital, and need for heart procedures. Patients who go to cardiac rehab also live longer than those who do not. Now that is motivation!

Cardiac rehab has many parts, including education, dietary counselling, exercise, stress management, support and heart pill optimization. If there are some parts in which you are not interested, talk to your cardiac rehab provider on how to tailor the program for you.

We would encourage you to at least go to an initial cardiac rehab session to share your goals, because the cardiac rehab team is there to create a rehab program that matches your interest (e.g., maybe you want to have a bit more vitality to spend time with grandchildren or work towards going on a trip you have always wanted to take). The cardiac rehab team is always open to suggestions on how to help you be more engaged and get what you are looking for out of participation.

20. Too long to get referred into the program

Unfortunately, there are many people like you that need cardiac rehab, so sometimes we have a back log in our programs. We regret this and work very hard to get patients started as soon as we are able. Patients who go to cardiac rehab live longer than those who do not, so we need to rectify the situation.

If you have contact information for the cardiac rehab program, contact them to confirm they have your referral information and find out when you can get started. If you do not, ask the provider you think referred you to confirm they did. If you were referred by someone at the hospital and were not sure who, you could ask your primary care provider to refer you.

If you have to wait to start cardiac rehab, you can start this heart patient education program available free online here: https://www.healtheuniversity.ca/en/cardiaccollege/Pages/default.aspx, accessed on 8 March 2023. You will find out how to start managing your risk factors, and exercise at a low level safely, until you can get started in your program.

It is still important for you to start your program when you do get the opportunity, no matter how much time has passed. Even if months have passed since your heart event, participating in cardiac rehab results in many benefits, such as better quality of life (you will have more energy and be able to do the things you want to do), less repeat visits to the hospital and need for heart procedures, and patients who go to cardiac rehab live longer than those who do not.

If you have to go back to work by the time they call you to start, ask the program if they have a home-based option. It may be possible for you to get your cardiac rehab through phone or video calls with your cardiac rehab team, after going for a few sessions to get oriented.

21. Prefer to exercise alone

I can be introverted and prefer to exercise on my own. Sounds like you might be the same way. Cardiac rehab participation brings so many health benefits, so please do not let this deter you from enrolling. Talk to your cardiac rehab program contact and let them know your preferences. Cardiac rehab programs are individually tailored to meet your needs. They may be able to give you 1-1 sessions or have other suggestions so you can still reap all the benefits of the programs.

22. Here, are the other barriers of concern for you that you can discuss with your healthcare providers
